# Development of a Photo-Crosslinking, Biodegradable GelMA/PEGDA Hydrogel for Guided Bone Regeneration Materials

**DOI:** 10.3390/ma11081345

**Published:** 2018-08-03

**Authors:** Yihu Wang, Ming Ma, Jianing Wang, Weijie Zhang, Weipeng Lu, Yunhua Gao, Bing Zhang, Yanchuan Guo

**Affiliations:** 1 Key Laboratory of Photochemical Conversion and Optoelectronic Materials, Technical Institute of Physics and Chemistry, Chinese Academy of Sciences, Beijing 100190, China; wyh8632@hotmail.com (Y.W.); maming@mail.ipc.ac.cn (M.M.); wangjianing@mail.ipc.ac.cn (J.W.); zhangweijie@mail.ipc.ac.cn (W.Z.); luweipeng@mail.ipc.ac.cn (W.L.); yhgao@mail.ipc.ac.cn (Y.G.); 2 School of Chemical Sciences, University of Chinese Academy of Sciences, Beijing 100049, China; 3 Hangzhou Branch of Technical Institute of Physics and Chemistry, Chinese Academy of Sciences, Hangzhou 310018, China

**Keywords:** gelatin, hydrogel, GelMA, PEGDA, photo-crosslinking

## Abstract

Gelatin-based hydrogel, which mimics the natural dermal extracellular matrix, is a promising tissue engineering material. However, insufficient and uncontrollable mechanical and degradation properties remain the major obstacles for its application in medical bone regeneration material. Herein, we develop a facile but efficient strategy for a novel hydrogel as guided bone regeneration (GBR) material. In this study, methacrylic anhydride (MA) has been used to modify gelatin to obtain photo-crosslinkable methacrylated gelatin (GelMA). Moreover, the GelMA/PEGDA hydrogel was prepared by photo-crosslinking GelMA and PEGDA with photoinitiator I2959 under UV treatment. Compared with the GelMA hydrogel, the GelMA/PEGDA hydrogel exhibits several times stronger mechanical properties than pure GelMA hydrogel. The GelMA/PEGDA hydrogel shows a suitable degradation rate of more than 4 weeks, which is beneficial to implant in body. In vitro cell culture showed that osteoblast can adhere and proliferate on the surface of the hydrogel, indicating that the GelMA/PEGDA hydrogel had good cell viability and biocompatibility. Furthermore, by changing the quantities of GelMA, I2959, and PEGDA, the gelation time can be controlled easily to meet the requirement of its applications. In short, this study demonstrated that PEGDA enhanced the performance and extended the applications of GelMA hydrogels, turning the GelMA/PEGDA hydrogel into an excellent GBR material.

## 1. Introduction

Hydrogels based on proteins or polysaccharide have been widely studied on account of their particular physical properties, excellent biocompatibility, and various composition [1,2]. In the past few decades, numerous hydrogels have been developed based on natural and/or synthetic materials [3,4,5], using various kinds of crosslinking methods such as chemical, physical, and free radical, for different biomedical applications [1,6,7,8], including tissue engineering scaffolds, wound dressing, drug delivery, artificial blood vessel, tissue regeneration, etc. [9,10,11,12].

Gelatin, the hydrolysis product of collagen, has similar amino acid composition to that of collagen, and it is without immunogenicity, which makes it one of the most useful biomaterials for tissue engineering [4,8,13]. According to the properties of gelatin, it can form a physically crosslinked hydrogel at room temperature, which restores the triple helical structure, and it is similar to collagen. However, the hydrogel is soluble in water at body temperature (37 °C). Therefore, the applications of the gelatin hydrogel in vivo are limited, due to its poor mechanical properties, rapid degradation rate, and low transition temperatures. To obtain suitable mechanical strength and stable gelatin hydrogel, various chemical crosslinking methods have been used, such as glutaraldehyde [14,15] and diisocyanate [16,17]. However, most of the chemical crosslinkers are toxic, and their use as cell-laden matrices for tissue engineering application is limited.

Van Den Bulcke et al. [18] developed a method to modify gelatin with methacrylic anhydride (MA) and obtained photocrosslinkable gelatin derivatives named gelatin methacrylamine (GelMA). Since then, GelMA has been widely studied as a biomaterial with attractive properties [19,20]. GelMA hydrogel is prepared by the photocrosslinking method, which has the advantages of an injectable, mild crosslinking condition and low cytotoxicity [1]. Several studies have demonstrated that GelMA hydrogel is suitable for both two-dimensional cell seeding and three-dimensional cell encapsulation [20,21,22] and is applicable to different manufacture technology, such as micromolding [23], self-assembling [23], microfluidics [23], bioprinting [24], and biotextiles [25]. The stiffness of GelMA hydrogel can be adjusted limitedly by controlling the degree of crosslinking [26], because the active groups on the gelatin chains, which can react with MA, are less than 5% of total amino acids. Moreover, the steric hindrance is increased with the degree of crosslinking, which hinders the crosslinking reaction.

Pure GelMA hydrogel is a good biodegradable material, however, when it is used as a GBR (Guided Bone Regeneration) material; its long gelation time, low mechanical strength, short degradation time, and high swelling rate restrict its applications. To be an eligible GBR material, a hydrogel must possess short gelation time to reduce operating time, suitable mechanical strength and degradation time to maintain space for bone reconstruction, and low swelling rate to reduce wound pressure to avoid inflammation. In this study, poly(ethylene glycol)diacrylate (PEGDA) was added into pre-polymer solution to increase the degree of crosslinking and inhibit the biodegradation rate. The PEGDA that we adopted is a micromolecule (<500 Da) with double active groups, which increases the chance of crosslinking in solution. The influence of PEGDA on gelation time, structure, stiffness, degradation, diffusion, and biocompatibility was investigated. Mouse osteoblasts were seeded on the hydrogel surfaces using the photocrosslinking method. The behaviors and fates of osteoblasts in hydrogel were studied using Calcein-AM/PI staining.

## 2. Materials and Methods

### 2.1. Materials

Gelatin (Type B from bovine bone, average molecular weight 80,000 Da) was obtained from Dongbao Bio-tech (Baotou, China). Methacrylic anhydride (MA), poly (ethylene glycol) diacrylate (PEGDA), 2-Hydroxy-1-(4-(hydroxyethoxy) phenyl)-2-methyl-1-propanone (Irgacure 2959), and deuterium oxide were purchased from Sigma-Aldrich (St. Louis, MO, USA). Collagenase Type I, FITC-BSA were purchased from Solarbio (Beijing, China). MC3T3-E1 (Mouse osteoblast cell line, 6 passages), fetal bovine serum, Alpha Modification Eagle Medium (α-MEM), and PBS buffer (pH 7.4) were purchased from Union Hospital (Beijing, China). The live/dead assay kit was purchased from ABcam (Britain, UK). All other reagents and solvents were of reagent grade.

### 2.2. Synthesis of Mechacrylated Gelatin (GelMA)

Methacrylated gelatin was synthesized according to the previously reported method [18]. Briefly, 10 g bovine bone gelatin was dissolved in 100 mL of phosphate-buffered saline at 60 °C for 60 min until totally dissolved. Then, 6 mL methacrylic anhydride was added to the gelatin solution at a rate of 0.5 mL min^−1^ and allowed to react for 3 h under stirred condition at 50 °C. The degree of methacrylation was affected by varying the amount of MA. Double distilled water was added into the mixture to stop the reaction. The solution was dialyzed against distilled water using 12–14 kDa dialysis tubing for 1 week. The solution was lyophilized and stored at −80 °C for further use.

### 2.3. Preparation of Hydrogel

GelMA/PEGDA hydrogels were prepared by photo-polymerization of two prepolymer (GelMA and PEGDA) at different weight ratios in aqueous solution with an initiator I2959 0.1% (*w*/*v*), which is shown in Table 1. The lyophilized GelMA was sterilized by ethylene oxide, and PEGDA and I2959 were dissolved in PBS buffer and filter-sterilized through 0.22 μm filter (produced by Millipore, Burlington, MA, USA). The mixed solution was placed into mold and exposed to 365 nm UV light (purchased from Haosifa Co., Ltd., ShenZhen, China, 90 mw/cm^2^) for 10 min at room temperature. The hydrogel was then taken out from mold and soaked in PBS buffer for 24 h to swell fully and remove the toxic residues.

### 2.4. NMR Spectra of GelMA

^1^H NMR was used to determine the methacrylation degree of free amine group in GelMA sample [26]. The method was reported as follows: 30 mg GelMA was dissolved in 1 mL D_2_O to get clear solution. The spectrum was obtained from Advance Bruker 400 M spectrometer. The methacrylation degree of GelMA was calculated as follows:

$$\mathrm{Methacrylation}\;\mathrm{degree}\;\left(\%\right)=\frac{\mathrm{Number}\;\mathrm{of}\;\mathrm{methacrylate}\;\mathrm{groups}}{\mathrm{Number}\;\mathrm{of}\;\mathrm{amine}\;\mathrm{group}\;\mathrm{on}\;\mathrm{unreacted}\;\mathrm{polymers}}\times 100$$

### 2.5. Scanning Electron Microscope Analysis

The GleMA/PEGDA hydrogel and GelMA hydrogel were immersed in PBS for 24 h at 37 °C before lyophilization and then cut into pieces by scalpel. The pore diameter and wall thickness were analyzed by Image J software (version 1.48u, National Institutes of Health, Bethesda, MD, USA). More than 30 pores were measured manually for each sample.

### 2.6. Swelling Ratio

The hydrogel was immersed in PBS for 24 h at 37 °C, and its swelling weight *W_s_* was measured. Then, the hydrogel was lyophilized to obtain dry weight *W_d_*. The swelling degree was calculated as following equation [27]:

$$\mathrm{Swelling}\;\mathrm{ratio}=\frac{Ws-Wd}{Ws}$$

### 2.7. Hydrogel Diffusivity

The water diffusivity of hydrogel for 1 h was measured according to the study reported. The hydrogel was cut into cylinder 10 mm in diameter and 4 mm high, then lyophilized. The lyophilized hydrogel was soaked in PBS at 37 °C. The absorbed water at 1 h for each hydrogel (*W*_1_) and equilibrium state (*W_e_*) was measured. L is the thickness of the hydrogel.

$$\frac{W_{1}}{W_{e}}=\frac{4}{\sqrt{\pi }}\times {\left(\frac{D\times t}{L^{2}}\right)}^{2}$$

Nutrient substance diffusion in the hydrogel was studied using the one-dimensional diffusion model [26]. Briefly, the hydrogel was cut into cylinders, then immobilized in a mold. 200 μL of 1% FTIC-BSA solution was dropped on the upper surface of hydrogel and washed 3 times with PBS to remove the fluorescent solution on the surface after 10 min. The fluorescence microscope (Leica, DMI6000B, Heidelberg, Germany) was used to observe the cross-section of the hydrogel.

### 2.8. Degradation by Collagenase

The hydrogels were incubated in 15 mL Eppendorf tubes with 5 mL PBS with 2 U mL^−1^ collagenase type I solution at 37 °C for 4 weeks. The collagenase solutions were replaced by fresh ones every 2 days to maintain constant enzyme activity. At different times, the sample was removed from collagenase solution and washed twice with sterile deionized water, lyophilized, and weighted [27]. The degradation rate was calculated using equation:

$$\mathrm{Degradation}\;\mathrm{Rate}\;\left(\%\right)=\frac{w_{0}-w_{t}}{w_{0}}\times 100\%$$

### 2.9. Compressive Mechanical Properties

The mechanical properties of GelMA/PEGDA hydrogels were measured using a universal testing machine (Instron 5960, Norwood, MA, USA) at a rate of 0.1 mm min^−1^ at 25 °C. The hydrogel samples were cut into cylinder 10 mm in diameter and 4 mm high and kept in PBS buffer for swelling for 24 h before testing.

### 2.10. 2D Cell Culturing

The hydrogel was prepared in the 24 well cell culture plate, then washed with PBS and α-MEM twice. The MC3T3-E1 with 2.0 × 10^4^ cells was seeded on each hydrogel surface. The Live-Dead Cell Staining Kit was used to evaluate cell proliferation after 1, 3, and 7 days of culture. The staining steps were as follows: Firstly, 5 μL Calcein-Am and 15 μL PI were added into 5 mL assay buffer to prepare staining solution; secondly, the cell culture medium was removed and the cells were washed by assay buffer twice; thirdly, 100 μL staining solution was added per well, and the cells were incubated for 30 min at 37 °C; finally, the fluorescence microscope (Leica, DMI6000B) was applied to observe the staining image, and the Image J software was used to count the number of live and dead cells.

### 2.11. Influence Factor of Gelation Time

According to the reaction mechanism, the concentrations of GelMA, PEGDA, and I2959 were regarded as the main influencing factors for gelation time, as long as UV source was fixed. Up to this point, there had been no existing products or methods with which to measure the gelation time caused by photo-initiation directly. So, we can measure the gelation time by adopting a physical observation as follows: Firstly, we got a coarse gelation time range of each gelatin by preliminary experiment. Then, we prepared a series of reaction mixtures for each sample in vials, treated them with UV, and inclined the vials to estimate whether phase transformed every minute.

### 2.12. Satistical Analysis

All results in this work were expressed as mean ± standard deviation. The GraphPad Prism version 7 (GraphPad Software, San Diego, CA, USA) was used for statistical analysis. Differences between group means were analysed with Student’s T test, and the level of significance was set at *p* < 0.05. The cell viability was analysed by Image J software.

## 3. Results and Discussion

### 3.1. Methacrylation of Gelatin

**Figure 1:**
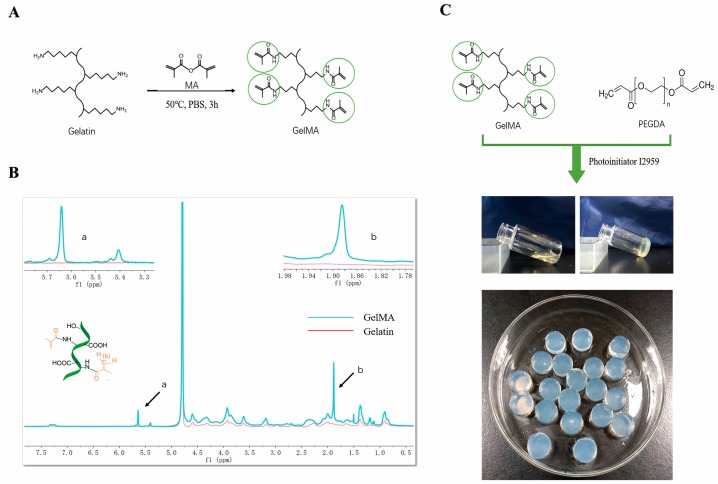
(**A**) Synthesis mechanism of GelMA; (**B**) the ^1^HNMR spectra of the GelMA (a, δ = 5.4 and 5.6 ppm; b, δ = 1.87 ppm); peaks at 5.4 ppm and 5.6 ppm correspond to two H methacrylic double bonds, while the peak at 1.87 ppm corresponds to the methyl group of methacrylic acid; (**C**) GelMA/PEGDA solution before and after 5 min UV treatment.

The method of preparation of GelMA was first reported by Van Bulcke et al. [18]. The reaction mechanism was displayed in Figure 1A. Briefly, methacrylic anhydride reacted with reactive amine and hydroxyl groups of amino acid residues to introduce unsaturated bond on gelatin molecular chain [28,29]. Thus, GelMA can be crosslinked via free radical photopolymerization in aqueous solution with photoinitiator. The degree of methacrylation can be controlled by the amount of methacrylic anhydride.

The degree of methacrylation of gelatin used in this study was 71.78% measured by ^1^H NMR spectrum. Figure 1B shows new signals appear at δ = 5.4 ppm and δ = 5.6 ppm in the spectrum of GelMA, which were the peaks of the acrylic protons of methacrylic functions; the peak at 1.87 ppm corresponds to the methyl group of methacrylic acid, and the peak at δ = 7.3 ppm represents the aromatic amino acid residues of gelatin.

### 3.2. Morphology of GelMA/PEGDA Hydrogel

**Figure 2:**
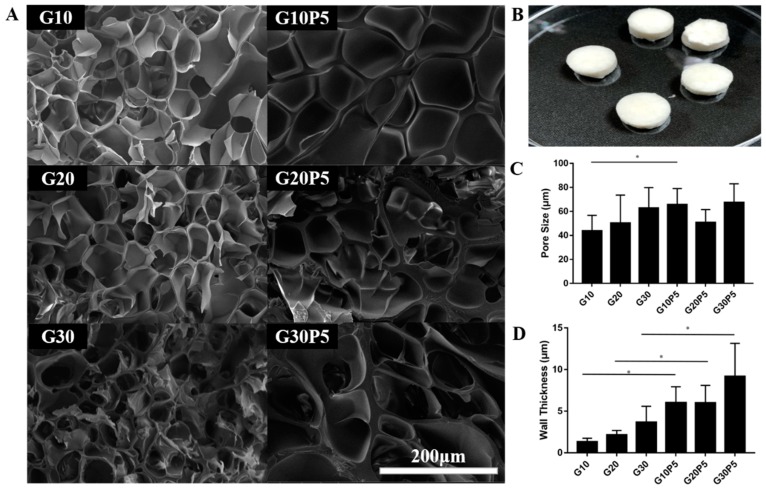
(**A**) SEM images of cross-section of GelMA hydrogel and GelMA/PEGDA hydrogel; (**B**) the Hydrogel was Freeze-dried after fully swelling; (**C**) the average pore size counted by Image J based on SEM images; (**D**) the average wall thickness counted by Image J based on SEM images (* *P* < 0.05).

GelMA/PEGDA hydrogel was prepared after the mixing of GelMA and PEGDA solution and photopolymerized with UV treatment (Figure 1C). The solution was free-flowing before UV treatment and turned into a gel phase after crosslinking. The hydrogel was fully swelling in PBS before lyophilization (Figure 2B), measured by SEM.

Although the interior structure of hydrogel maybe different from the natural state before lyophilization, it is still a useful method for investigating the interior 3D structure of hydrogel. Figure 2A shows the GelMA hydrogel and GelMA/PEGDA hydrogel cross-section images as comparison. The pore diameter (Figure 2C) of G10 was 43.79 ± 12.89 μm, and G10P5 was 65.56 ± 13.45 μm, which was significantly larger than G10 (*p* < 0.05). The pore diameter sof G20 and G30 were similar to G20P5 and G30P5; they showed no significant change. The PEGDA reacted with GelMA in the system and significantly increased the pore diameter that was very suitable for human cell growth in guide bone regeneration application. As shown in Figure 2D, the thickness of pore wall from G10 to G30P5 was 1.33 ± 0.42, 2.15 ± 0.54, 3.68 ± 1.92, 6.02 ± 1.93, 6.00 ± 2.11, and 9.17 ± 3.98, respectively. The wall thickness of hydrogel was significantly increased (*p* < 0.05) when PEGDA was added into reaction system, which enabled the hydrogel with better mechanical properties. In addition, an increase of wall thickness can lead to a decrease of pore density, which results in an increase of average pore diameter per unit volume, as shown in Figure 2A.

### 3.3. Swelling Ratio of GelMA/PEGDA Hydrogel

**Figure 3:**
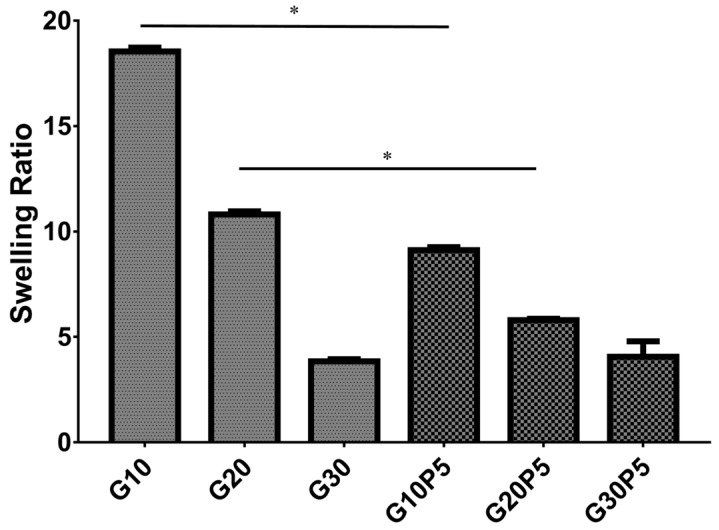
Swelling ratio of GelMA and GelMA/PEGDA hydrogels in PBS solution at room temperature (* *P* < 0.05).

Swelling ratio of hydrogel was an essential factor for tissue engineering application. The swelling ratios of GelMA and GelMA/PEGDA hydrogel were calculated as shown in Figure 3. The swelling ratios of G10, G20, G30, G10P5, G20P5, and G30P5 were 18.33 ± 0.19, 10.81 ± 0.15, 3.83 ± 0.11, 9.11 ± 0.14; 5.79 ± 0.07, and 4.06 ± 0.74, respectively. The swelling ratio of hydrogel decreased significantly (*p* < 0.05) with the amount of PEGDA added. This was because the PEGDA could increase the degree of crosslinking. The high crosslinking degree improved the stiffness of hydrogel and led to low swelling ratio [1,26], which made the hydrogel suitable for implanted material at low wound pressure.

### 3.4. Diffusivity of Hydrogel

**Figure 4:**
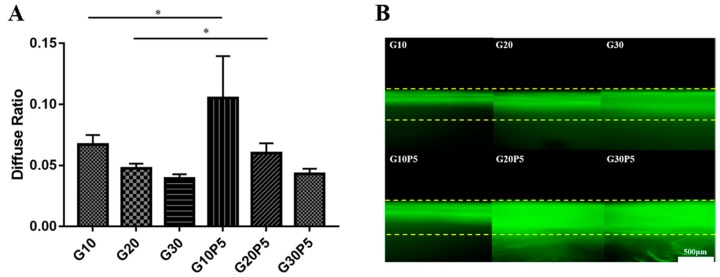
(**A**) The diffusion coefficient of water for 1 h; (**B**) the FITC-BSA one-dimensional diffusion in hydrogel (* *P* < 0.05).

As shown in the Figure 4A, PEGDA could react with GelMA and increase the water diffusivity of hydrogel as compared to the hydrogel without PEGDA. The D values of G10, G20, G30, G10P5, G20P5, and G30P5 were 6.71 ± 0.78 (×10^−2^), 4.76 ± 0.38 (×10^−2^), 3.93 ± 0.36 (×10^−2^), 10.54 ± 3.43 (×10^−2^), 6.00 ± 0.82 (×10^−2^), and 4.32 ± 0.41 (×10^−2^), respectively. According to these data, the diffusion resistance of G10P5 and G20P5 was reduced significantly (*p* < 0.05) compared with G10 and G20, when PEGDA was added into reaction system. However, there was no significant difference between G30 and G30P5, and this may be caused by the similar pore diameter of G30 and G30P5.

Figure 4B shows the cross-section of the one-dimensional diffusion of FITC-BSA in the hydrogel. The depth represented the diffusion intensity, which simulated the nutrient substance transmission in hydrogel, which is an important factor for tissue engineering material. The PEGDA increased the crosslinking degree and resulted in larger pore diameter, which enabled the macromolecular substances to be more easily transported into the hydrogel.

### 3.5. Biodegradation of Hydrogel In Vitro

**Figure 5:**
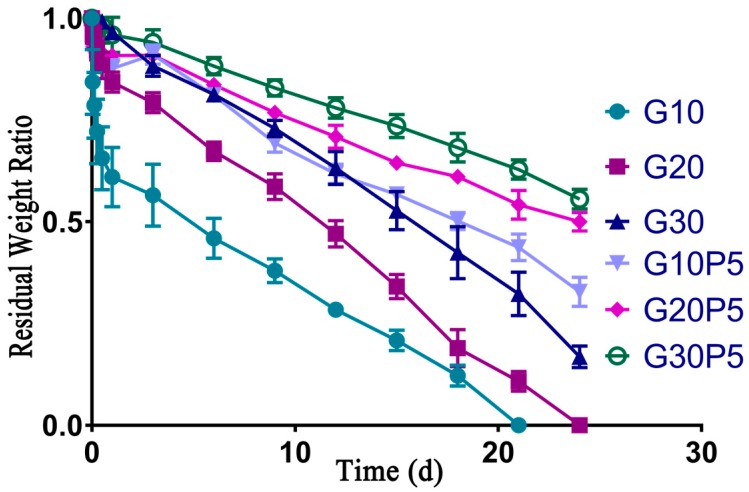
Biodegradation of GelMA and GelMA/PEGDA hydrogel at 37 °C in collagenase type I solution.

The degradation rate of hydrogel in PBS solution without collagenase was very slow, and especially the GelMA/PEGDA hydrogel was nearly non-degradable. The Figure 5 shows the biodegradation results of GelMA and GelMA/PEGDA hydrogel. All the hydrogels were soaked in PBS for 24 h to fully swell and remove the residues before test. The G10 and G20 were totally degraded after 4 weeks; however, the hydrogel with PEGDA could maintain its shape, and the residual weight ratios of G20P5 and G30P5 were more than 50% after 4 weeks. The hydrogel with PEGDA degraded more slowly than the pure GelMA hydrogel, as the PEGDA improved the crosslinking degree and made the 3D structure of hydrogel more complicated. Thus, the GelMA/PEGDA hydrogel needed long time to degrade in vitro, and this overcame the shortcoming of pure GelMA hydrogel.

### 3.6. Compression Test

**Figure 6:**
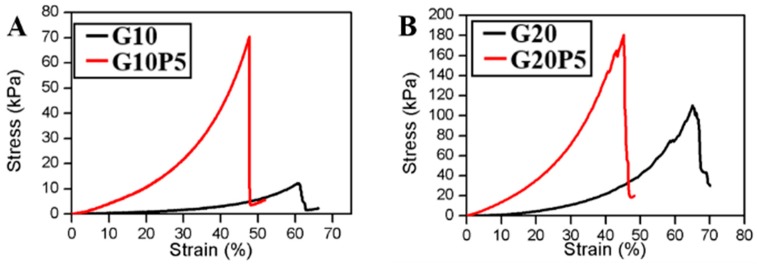

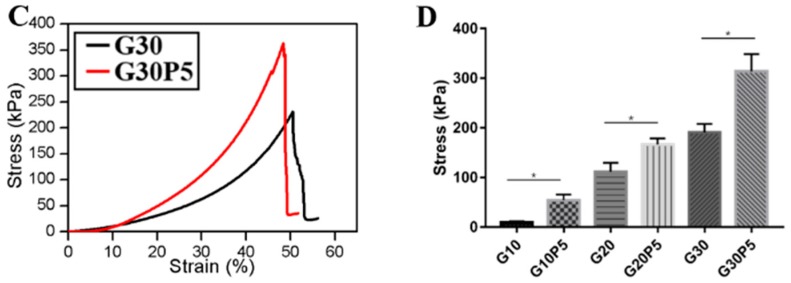
(**A**–**C**) Compression stress-strain curves of GelMA and GelMA/PEGDA hydrogel; (**D**) the maximum compressive stress of different hydrogels (* *P* < 0.05).

As shown in Figure 6A–C, the GelMA/PEGDA hydrogel had higher compressive stress than that of pure GelMA hydrogel. The stress of G10P5 was 70.6 kPa, which was almost 6 times G10 (12.1 kPa). The strain of G10P5 was 47.9% less than G10 (60.8%). The PEGDA significantly (*p* < 0.05) increased the compressive stress compared with the hydrogel without PEGDA, as shown in Figure 6D. These results were attributed to the high degree of crosslinking interaction between GelMA and PEGDA network. The results were also proved by the SEM image mentioned before; the high stress GelMA/PEGDA hydrogel had thicker walls. However, the higher concentration of GelMA and GelMA/PEGDA led to higher crosslinking density, which increased the hydrogel stiffness and became more fragile.

### 3.7. In Vitro Cell Culture

**Figure 7:**
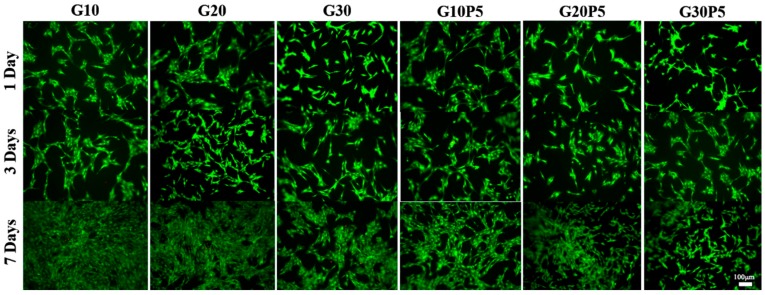
Live/dead staining of MC3T3-E1 cultured on the surface of hydrogel for 1 day, 3 days, and 7 days. (Green: Live cells; red: Dead cells).

Live/dead cell staining method was used to investigate the cell viability of MC3T3-E1 cultured on the surface of GelMA and GelMA/PEGDA hydrogel for 1, 3, and 7 days. As shown in Figure 7, almost all of the cells were alive after 1, 3, and 7 days culture for all the samples; the viability was greater than 99% analyzed by Image J software. The images manifested that the photo-crosslinking treatment of hydrogel was nontoxicity to cells. The hydrogel with PEGDA showed no difference from the pure GelMA hydrogel, suggesting the PEGDA did have a toxic effect on cell viability. The GelMA/PEGDA hydrogel showed good biocompatibility and could be used for cell encapsulation.

### 3.8. Gelation Time Study

**Figure 8:**
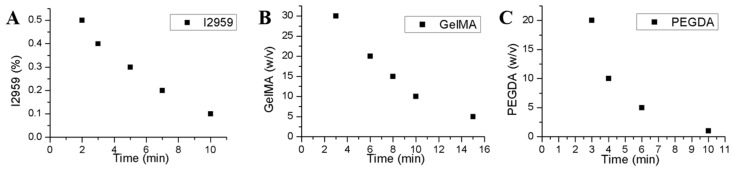
(**A**) The impact of I2959 concentration on gelation time, when concentration of GelMA was 10% w/v; (**B**) the impact of GelMA concentration on gelation time, when concentration of I2959 was 0.1% w/v; (**C**) the impact of PEGDA concentration on gelatin time, when concentation of I2959 was 0.1% and GelMA was 10% *w*/*v*.

The gelation time was studied by changing the mass of I2959, GelMA, and PEGDA. Figure 8A,B indicated that gelation time could be reduced by increasing the photoinitiator I2959 or prepolymer GelMA concentration. Figure 8C demonstrated that only a small quantity of PEGDA can drastically reduce the gelation time. Thus, the gelation time could be controllable adjustment according to application requirement.

## 4. Conclusions

This study synthesized a new biocompatible and biodegradable GelMA/PEGDA hydrogel by UV photo-crosslinking. The properties of morphology, swelling, diffusion, degradation, and mechanical and cell viability were systematically studied. The GelMA/PEGDA hydrogel had a higher mechanical strength, longer degradation time, faster diffusion rate, and lower swelling rate than the pure GelMA hydrogel. In vitro cell culture experiments—mouse osteoblasts MC3T3-E1 culture on the GelMA/PEGDA surface—showed high viability, adhesion, and proliferation. Moreover, the gelation time could be adjusted and reduced the operating time. In summary, this study demonstrated that PEGDA can enhance the performance and extend the applications of GelMA hydrogels as a promising GBR material.

## Figures and Tables

**Table 1:** Composition of hydrogels.

Samples	Abbreviation	GelMA % (*w*/*v*)	PEGDA % (*w*/*v*)
GelMA 10%	G10	10	---
GelMA 20%	G20	20	---
GelMA 30%	G30	30	---
GelMA 10% PEGDA 5%	G10P5	10	5
GelMA 20% PEGDA 5%	G20P5	20	5
GelMA 30% PEGDA 5%	G30P5	30	5
